# Primary high-grade neuroendocrine carcinoma with positive steroid hormone receptors arising in the inguinal skin: A case report of An exceedingly rare entity

**DOI:** 10.1097/MD.0000000000036624

**Published:** 2023-12-15

**Authors:** Ning Zhou, Fanrong Wang, Li Yang, Qin Wang, Jun Liu, Ying Chen

**Affiliations:** a Department of Pathology, Sichuan Mianyang 404 Hospital, Mianyang, Sichuan Province, China; b Department of Pathology, The People’s Hospital of Santai County, Mianyang, Sichuan Province, China; c Department of Breast surgery, Sichuan Mianyang 404 Hospital, Mianyang, Sichuan Province, China; d Department of Pathology, Guiqian International General Hospital, Guiyang, Guizhou Province, China.

**Keywords:** ectopic breast tissue, inguinal, mucinous, neuroendocrine carcinoma of the skin, primary cutaneous neuroendocrine

## Abstract

**Introduction:**

Neuroendocrine tumors usually arise from the gastrointestinal and pulmonary tracts and rarely from the skin. We report a unique case of high-grade neuroendocrine carcinoma with positive steroid hormone receptors in the primary skin of the groin.

**Case presentation:**

A 79-year-old female presented with a lump in her left inguinal region for 15 years that grew gradually. The tumor cells were arranged in sheets, solid nests, and bands within a rich network of thin-walled capillaries. Mucin was abundant in the stroma, and the tumor cells exhibited high-grade lesions, significant necrosis, and frequent mitosis, with small scattered foci of low-grade components. Immunohistochemistry revealed that the tumor cells diffusely and strongly expressed cytokeratin, synaptophysin, chromogranin A, GATA3, CAM5.2, and estrogen and progesterone receptors; partially expressed AR and GCDFP15.

**Diagnosis:**

Based on pathological morphology, and immunohistochemical staining, it was confirmed as Primary high-grade neuroendocrine carcinoma with positive steroid hormone receptors arising in the inguinal skin. The patient underwent resection of the inguinal tumor and left inguinal lymph node dissection.

**Interventions:**

The patient has been followed up for 16 months and has not undergone further examinations or received additional treatment. There is no evidence of tumor recurrence at the site of the original surgical resection, and the patient general condition is satisfactory.

**Conclusions:**

The morphology of this tumor is unique and previously unreported, further expanding the possible pathogenesis and histological morphologies of this tumor type.

## 1. Introduction

Neuroendocrine tumors (NETs) are a heterogeneous group of rare tumors derived from peptidergic neurons and specialized neuroendocrine cells capable of secreting various peptides or amines. These cells may be abundantly present in endocrine tissues or diffusely in tissues of the digestive or respiratory system. NETs commonly arise from the gastrointestinal and pulmonary tracts and rarely from the skin. Only 2 subtypes of primary cutaneous neuroendocrine carcinoma were recognized in the 2018 World Health Organization classification of skin tumors: Merkel cell carcinoma (MCC; also known as primary cutaneous neuroendocrine carcinoma) and endocrine mucin-producing sweat-gland carcinoma (EMPSGC).^[1]^ Here, we report a case of high-grade neuroendocrine carcinoma with positive hormone receptors. Its unique morphology differs from previous reports on this type of tumor, and the origin of this type of lesion has not been established.

## 2. Case presentation

### 2.1. Case characteristics

The patient was a 79-year-old female with a left inguinal mass for 15 years but did not take it seriously or receive further examination. The mass gradually grew and was diagnosed as a malignant tumor by puncture biopsy in a local hospital 5 days before presentation at our hospital. Nil significant medical or family history. Further examination at our hospital revealed a huge, hard mass in the left inguinal area with visible ulceration on the tumor surface. Ultrasound showed a suspicious solid nodule on the uterine wall, indicating the possibility of uterine fibroids. No abnormalities were found in other organs. Computed tomography revealed no other tumor lesions in other body parts. The patient underwent resection of the inguinal tumor and left inguinal lymph node dissection, which revealed poor tumor activity and invasion of the left inguinal ligament during surgery.

## 3. Pathological findings

**Figure 1.**
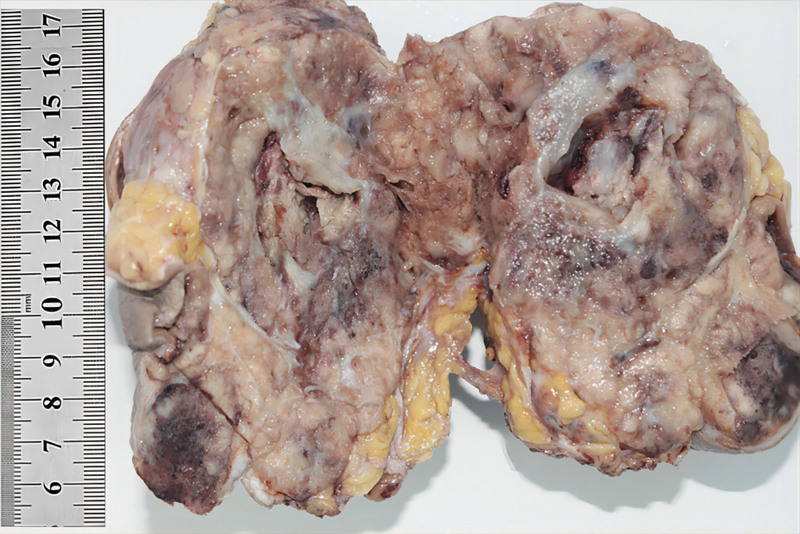
Neuroendocrine carcinoma. The tumor appears gray-white to gray-brown and shows cystic changes.

**Figure 2.**
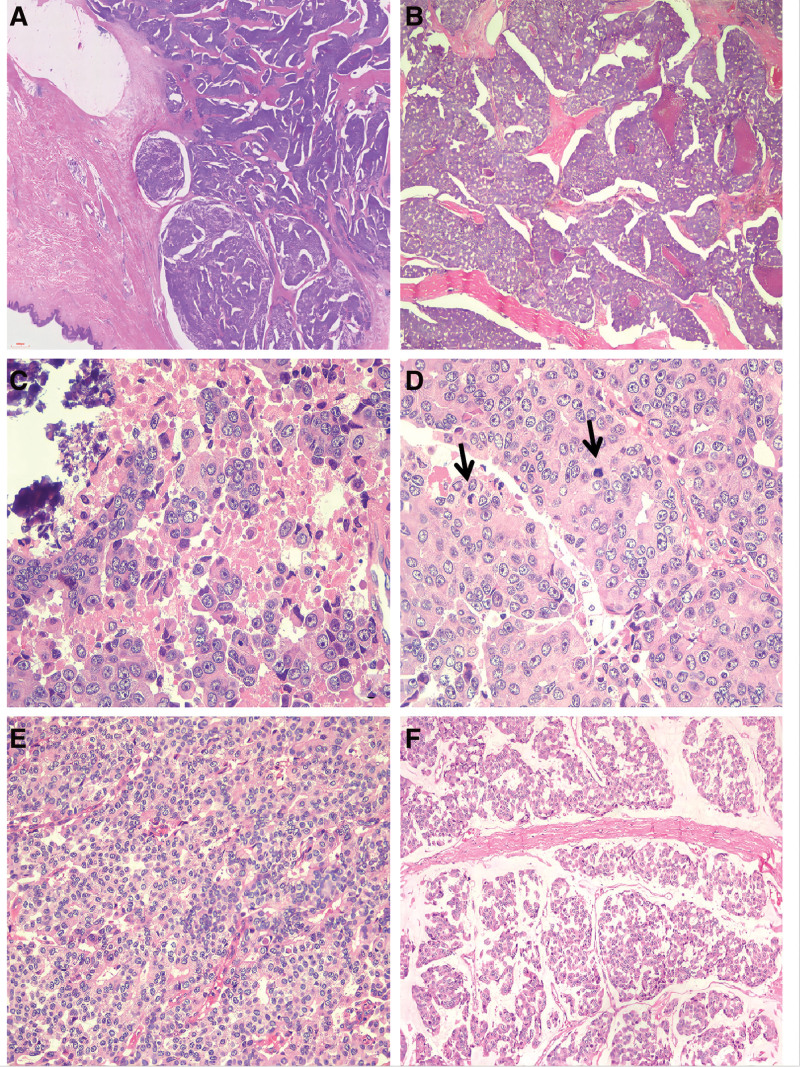
Neuroendocrine carcinoma images. Low-magnification microscope image showing that the tumor is located in the dermis and subcutaneous tissue, and the tumor cells are arranged in sheet-, solid nest-, and ribbon-like structures (A × 20). Low-magnification microscope image showing that the tumor has sieve-like and pseudoglandular structures formed by cells within the nests, with significant visible necrosis (B × 100). Cytologically, the tumor shows high-grade features with abundant cytoplasm; amphophilic, large round or oval nuclei with dispersed chromatin; prominent nucleoli; and evidence of necrosis and calcification (C × 400). The nucleoli are prominent, and mitosis is easily observed, as indicated by the arrow (D × 400). An area of low-grade neuroendocrine carcinoma, with cells having eosinophilic cytoplasm and small, uniform nuclei (E × 400). Tumor cell clusters or nests float in the mucinous stroma, resembling the pattern of breast mucinous carcinoma type B (F × 200).

**Figure 3.**
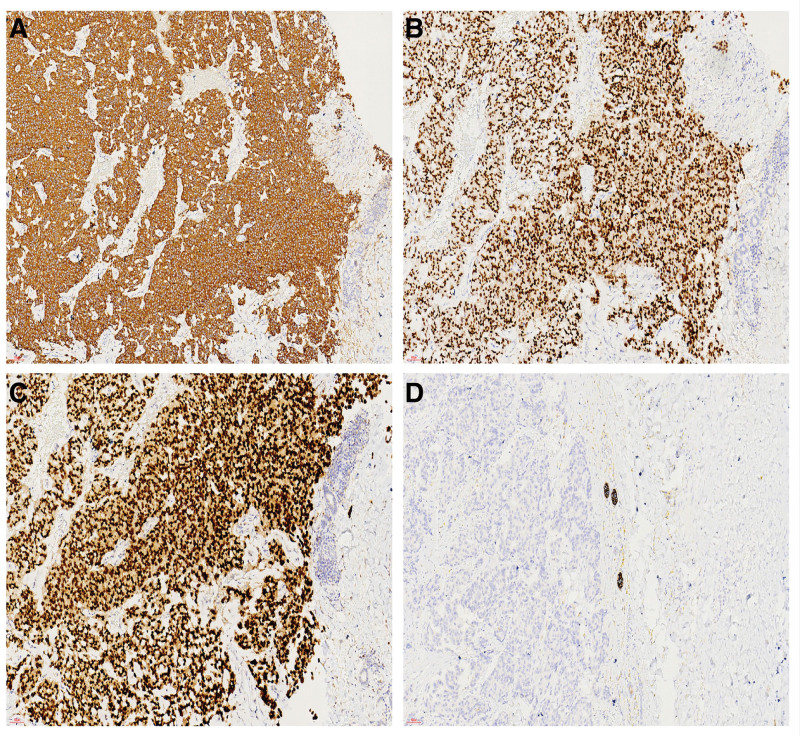
Immunohistochemistry showing diffuse positive expression of chromogranin A (A × 200). Immunohistochemistry showing almost 100% expression of ER in tumor cells (B × 200). Immunohistochemistry showing diffuse positive expression of GATA-3 (C × 200). Immunohistochemistry showing negative expression of CK7 (D × 200).

The left inguinal mass was a solid grayish-yellow and grayish-brown mass with skin measuring 12 × 9.4 × 8.5 cm(Fig. 1). Histologically, the tumor involved the dermis and subcutaneous soft tissue with visible ulceration on the skin surface. The tumor border was unclear, and infiltrative growth was observed. The tumor cells were arranged in sheets, solid nests, and band-like structures. Collagen fiber segmentation was observed around the nests, and some nests contained cells forming sieve-like and pseudoglandular structures. Significant necrosis and calcification were observed within the tumor cell nests (Fig. 2A and B). Cytologically, the tumor showed high-grade characteristics, with rich cytoplasm, eosinophilic or amphophilic. The nuclei of the cells were large, round, or oval, with dispersed chromatin and prominent nucleoli. Significant or atypical mitosis (23 mitoses/2 mm^2^) was observed (Fig. 2C and D). Scattered areas of small focal low-grade cells were seen therein (Fig. 2E). Abundant thin-walled capillaries were visible within and between the tumor nests. In some areas, the stroma was significantly mucinous, and tumor cell clusters or nests floated within the mucinous stroma, similar to mucinous breast type B carcinoma. This area accounted for approximately 40% of the tumor (Fig. 2F). Five inguinal lymph nodes were observed, 3 of which showed cancer metastasis. Immunohistochemistry showed that the tumor cells diffusely and strongly expressed cytokeratin, synaptophysin, chromogranin A, GATA3, and CAM5.2. Estrogen and progesterone receptors (ER and PR, respectively) were highly expressed at approximately 100%, and AR expression was also high at 80%. The expression level of gross cystic disease fluid protein 15 was approximately 15%. Furthermore, mammaglobin was focally expressed, while other antibodies, namely, cytokeratin7, 20, PAX8, CDX-2,WT1, p63, and Merkel cell polyomavirus large T-antigenwere negatively expressed (Fig. 3A–D). The Ki-67 index was approximately 30%.

## 4. Diagnosis

Based on pathological findings, a confirmed diagnosis of Primary high-grade neuroendocrine carcinoma with positive steroid hormone receptors arising in the inguinal skin was made. The patient has been followed up for 16 months and has not undergone further examinations or received additional treatment. There is no evidence of tumor recurrence at the site of the original surgical resection, and the patient general condition is satisfactory.

## 5. Discussion

NETs originating from the skin are very rare. MCC and EMPSGC have been well-established as primary cutaneous neoplasms with neuroendocrine differentiation.^[2]^ We report a unique case of high-grade neuroendocrine carcinoma with positive steroid hormone receptors in the primary skin of the groin and it exhibits an indolent biological behavior. To our best knowledge, this type of lesion has not been reported before. Its unique morphology differs from previous reports on this type of tumor, and the origin of this type of lesion has not been clearly established.

MCC cells exhibit high-grade cytomorphology with a high mitotic and Ki-67 proliferative index. Diagnosis involves recognizing the characteristic histological features, including a monotonous population of tumor cells with overlapping vesicular nuclei containing a smattering of small nucleoli in a background of finely granular or dusty chromatin. Nuclear molding, another characteristic of pulmonary small cell carcinoma, may also be a feature.^[2]^ MCC is characterized by positive CK20 staining; a paranuclear dot-like pattern. In this case, there was negative CD20 and Merkel cell polyomavirus large T-antigen expression but positive expression of hormone receptor-related markers, ruling out skin-derived MCC.

EMPSGC is a rare, low-grade, cutaneous adnexal carcinoma with neuroendocrine differentiation. It is believed to be similar to solid papillary carcinoma of the breast and also a precursor lesion of neuroendocrine-type mucinous sweat gland carcinoma (MSC) of the skin.^[3,4]^ The predilection site is the skin of the lower eyelid (55.6%), followed by the upper eyelid (36.5%), canthus (3.2%), but it has also been reported in the face and, rarely, in extra-facial locations.^[5]^ EMPSGC shows in situ growth or expanding invasion growth. Small amounts of intracellular and extracellular mucin are often identifiable on hematoxylin-eosin staining. Mitotic activity is low, with absence of tumor necrosis. EMPSGC can transform into mucinous carcinoma in the invasive phase and is associated with invasive neuroendocrine type MSC in 35.7% of cases. Further, EMPSGC-associated MSC recurs in approximately 12.3% of cases. However, metastasis is rare; only one locoregional metastasis out of 190 EMPSGC cases has been reported.^[6]^ Therefore, the extrafacial location, lack of in situ tumor component, and infiltrative growth in the present case rule out EMPSGC and neuroendocrine-type MSC.

Very rarely, NETs can arise from the skin as a low-grade lesion. Low-grade neuroendocrine carcinomas of the skin (LGNECS) was proposed in 2017 as a distinct third entity of primary cutaneous tumor with neuroendocrine differentiation, and this tumor was presumed to originate from the apocrine/eccrine apparatus.^[7]^ Histopathologically, it is a low-grade tumor and similar to that of NETs in other organs. On immunohistochemical examination, LGNECS frequently express ER, PR, AR, GCDFP15, mammaglobin, cytokeratin 7, and GATA3, as well as neuroendocrine markers.^[8]^ In this case, a markedly high-grade cytological morphology with marked necrosis and mitosis distinct from LGNECS was observed.

Three cases of primary cutaneous apocrine adenocarcinoma with neuroendocrine differentiation have been reported in recent years.^[9–11]^ The origin and classification of these tumors are still controversial. These tumors show common histopathology and immunohistochemistry characteristics with breast cancer, and they affect the pudendal or near the pudendal area, where mammary-like glands can be seen.^[12]^ Therefore, these tumors might originate from mammary-like sweat glands in the anogenital region. As they do not show any definite morphological findings of apocrine snouts, Goto et al^[8]^ suggest that they might also be LGNECS.

The origin of Primary high-grade neuroendocrine carcinoma with positive steroid hormone receptors arising in the inguinal skin remains uncertain, but we speculated that it might have originated from Ectopic breast tissue (EBT). EBT is an embryological abnormality resulting from the involutional failure of the mammary ridge, commonly called the milk line. The milk line starts from the axilla and extends inferior to the groin, leading to the possibility of EBT along this axis. Ectopic breast cancer accounts for 0.2% to 0.6% of all breast cancers.^[13]^ EBT in the inguinal region has been reported in previous literature.^[14]^ In this case, the tumor originated from the groin and grew for 15 years. Ultrasound and computed tomography scans did not detect any tumors in the breast, axilla, or other areas. The histological morphology and immunohistochemistry showed some similarities with breast cancer, with tumor cells showing almost 100% expression of breast-related markers such as ER, PR, and GATA3. Although EBT was absent in the tumor, we still speculated that it might have originated from EBT. As sweat glands and breast tissue share a common embryological origin and show similar immunohistochemical expression, we cannot completely rule out the possibility of sweat gland origin. Although the tumor mainly showed high-grade lesions, small scattered foci of low-grade lesions were also observed. Considering the tumor growth history, we speculated that it was a low-grade neuroendocrine carcinoma with high-grade transformation.

## 6. Conclusions

We report the first case of Primary high-grade neuroendocrine carcinoma with positive steroid hormone receptors from the skin of the groin. The tumor had a unique histological morphology different from previously reported primary cutaneous neuroendocrine carcinomas, further expanding the understanding of the onset sites and histological morphology of this tumor type. In our case, while the histology is predominantly high-grade, it exhibits an indolent biological behavior. This indicates that there may be differences, in terms of both biological behavior and tissue origin, between cutaneous primary neuroendocrine carcinomas that express hormone receptors and neuroendocrine carcinomas that do not express hormone receptors.

In this case, we did not find any ectopic breast tissue around the tumor, so whether it is related to ectopic breast needs more cases to further establish the origin and classification of this rare tumor.

## Acknowledgments

We would like to thank Editage (www.editage.cn) for English language editing.

## Author contributions:

**Conceptualization:** Ning Zhou, Fanrong Wang.

**Data curation:** Li Yang, Jun Liu, Ying Chen.

**Funding acquisition:** Ning Zhou.

**Writing – original draft:** Ning Zhou, Fanrong Wang.

**Writing – review & editing:** Li Yang, Qin Wang, Jun Liu, Ying Chen.
